# Processing Attenuating NPIs in Indicative and Counterfactual Conditionals

**DOI:** 10.3389/fpsyg.2022.894396

**Published:** 2022-06-10

**Authors:** Juliane Schwab, Mingya Liu

**Affiliations:** ^1^Institute of Cognitive Science, Osnabrück University, Osnabrück, Germany; ^2^Department of English and American Studies, Humboldt University of Berlin, Berlin, Germany

**Keywords:** conditionals, negative polarity items, counterfactual presupposition, conditional perfection, pragmatics, English

## Abstract

Both indicative and counterfactual conditionals are known to be licensing contexts for negative polarity items (NPIs). However, a recent theoretical account suggests that the licensing of attenuating NPIs like English *all that* in the conditional antecedent is sensitive to pragmatic differences between various types of conditionals. We conducted three behavioral experiments in order to test key predictions made by that proposal. In Experiment 1, we tested hypothetical indicative and counterfactual conditionals with the English NPI *all that*, finding that the NPI is degraded in the former compared to the latter. In Experiment 2, we compared hypothetical indicative conditionals and premise conditionals with the same NPI, again finding a degradation only for the former. Both results align with theoretically derived predictions purporting that hypothetical indicative conditionals are degraded due to their susceptibility to conditional perfection. Finally, Experiment 3 provides empirical evidence that comprehenders readily strengthen counterfactual conditionals to biconditionals, in line with theoretical analyses that assume that conditional perfection and counterfactual inferences are compatible. Their ability to still host attenuating NPIs in the conditional antecedent, by contrast, falls into place *via* the antiveridical inference to the falsity of the antecedent. Altogether, our study sheds light on the interplay between NPI licensing and the semantic and pragmatic properties of various types of conditionals. Moreover, it provides a novel perspective on the processing of different kinds of conditionals in context, in particular, with regard to their (non)veridicality properties.

## 1. Introduction

Natural language conditionals are known to host a range of pragmatic inferences in the form of implicatures and presuppositions. One particularly well-known case is the conversational implicature termed ‘conditional perfection’ (henceforth CP) in indicative conditionals, whereby a conditional proposition as in (1) may receive a logically biconditional interpretation (2) *via* the additional inference of (1-a) or (1-b) (Geis and Zwicky, 1971).

(1) If you mow the lawn, I'll give you $5.
  a. If you don't mow the lawn, I won't give you $5.
  b. Only if you mow the lawn, I'll give you $5.
(2) If and only if you mow the lawn, I'll give you $5.

The CP inference concerns the relationship between the antecedent and consequent proposition, such that the former is interpreted not just as sufficient, but as necessary and sufficient to bring about the consequent. However, pragmatic inferences of conditionals can also concern the status of the antecedent proposition itself, as evidenced by two other types of conditionals: In counterfactual conditionals like (3), the falsity of the antecedent proposition (3-a) is inferred as a presupposition or conversational implicature (Anderson, 1951; von Fintel, 1998; Iatridou, 2000; Ippolito, 2003; Leahy, 2018; Zakkou, 2020). The exact status of this inference has been a subject of debate, as is discussed below. Conversely, in premise conditionals (also termed factual conditionals, Iatridou, 1991), it is presupposed that the antecedent proposition is believed to be true by some salient discourse participants, e.g., by speaker A in (4) (Iatridou, 1991; Haegeman, 2003).

(3) If we had left at noon, we would have arrived in time for dinner.
  a. We didn't leave at noon.
(4) A: Anne is sick.
B: If Anne is sick, she won't come to the party.

In a recent theoretical analysis, Schwab and Liu (2022) propose that the pragmatics of these three types of conditionals has immediate consequences on their ability to embed attenuating negative polarity items (NPIs). NPIs are a set of natural language expressions whose distribution is restricted to negative and negation-related sentential contexts (5), including the antecedent of conditionals (e.g., Giannakidou, 1998; von Fintel, 1999). But although attenuating NPIs like *all that* are acceptable in counterfactual (6-b) and premise conditionals (6-c), they appear to be degraded in hypothetical indicative conditionals (6-a). In an argument we will lay out in detail below, Schwab and Liu stipulate that the degradation of hypothetical indicative conditionals is due to the presence of CP inferences. As premise conditionals cannot be perfected, they are considered immune to this effect. Finally, for counterfactual conditionals, it is assumed that the inference to the falsity of the antecedent can improve the conditional as an NPI licenser.

(5) The contract negotiations have^*^(n't) gone all that well.
(6) a. ?If the contract negotiations have gone all that well, Sue will take the job.
b. If the contract negotiations had gone all that well, Sue would have taken the job.
c. A: The contract negotiations have gone very well.
B: If the contract negotiations have gone all that well, Sue will take the job.

The predictions of this proposal have so far not been tested empirically. In the present article, we, therefore, use a series of behavioral experiments to verify key assumptions about the processing of the attenuating NPI *all that* in hypothetical indicative, counterfactual, and premise conditionals and determine to what extent comprehenders draw the relevant pragmatic inferences—CP and antecedent falsity—in the comprehension process. Our findings by and large support Schwab and Liu's theoretical analysis; however, they also raise novel questions about the respective contribution each of these inferences makes to the interpretation of conditionals and, consequently, the licensing of attenuating NPIs.

In the following, we first introduce the relevant theoretical background on CP and counterfactual inferences, before turning to the proposal by Schwab and Liu in Section 3. Sections 4–6 report three behavioral experiments. Section 7 concludes with a General Discussion.

## 2. Two Inferences in Conditionals

### 2.1. Antecedent Falsity

Counterfactual conditionals, marked as such by past perfect morphology on the antecedent and the modal verb *would* on the consequent in English, assume a counterfactual state of the world in the antecedent proposition and relate it with presumed consequences in the consequent proposition. Therein, the falsity of the antecedent has been treated as conversationally implicated (Anderson, 1951; Iatridou, 2000; Ippolito, 2003; Arregui and Biezma, 2016; Leahy, 2018) or presupposed (Zakkou, 2020) meaning component of the conditional. Advocates of the conversational implicature view have argued that antecedent falsity cannot be a presupposition given its apparent cancelability in so-called ‘Anderson conditionals’ like (7) (Anderson, 1951):

(7) If Jones had taken arsenic, he would have shown the same symptoms he actually shows. (So he took arsenic).

As the argument goes, if the counterfactual conditional in (7) were to presuppose that *Jones didn't take arsenic*, the speaker would be contradicting herself by subsequently asserting the opposite. Thus, antecedent falsity must be a cancelable implicature. Recently, however, Zakkou (2020) has argued that this does not present a conclusive argument against the presupposition view. She argues that presupposing *p* and asserting *p* are not governed by the same norms, such that by asserting *p*, a speaker is asking to add a proposition to the common ground as a mutually accepted belief, but by presupposing *p*, a speaker is merely asking to accept the proposition as a mutually agreed assumption for a given purpose. On this view, a speaker is not contradicting herself in Anderson conditionals: she accepts *not-p* as an assumption within the current context (thus presupposing the falsity of the antecedent), and subsequently asserts *p*, thereby clarifying that she accepts *p* as the actual state of the world. Additionally, in Anderson conditionals like (7) the consequent proposition provides a good reason for the speaker's conclusion that *p* must be true after all. Zakkou shows that this also explains why Anderson conditionals like (8), where the speaker's presupposition and assertion are compatible, are bad—after all, on what basis is the speaker coming to her conclusion? Although not discussed here, Zakkou's reasoning extends to other commonly held arguments in favor of the conversational implicature view (e.g., by Anderson, 1951; Iatridou, 2000; Ippolito, 2003; Arregui and Biezma, 2016; Leahy, 2018). In short, she argues that neither of these are conclusive cases against the presupposition view; we refer the reader to Zakkou (2020) for details.

(8) If Jones had taken arsenic, he would have shown the same symptoms he actually shows. (#So he didn't take arsenic).

Overall, the linguistic status of the inference to the falsity of the antecedent thus arguably remains in question. In any case, whether treated as conversational implicature or as presupposition, it is seen as salient inference in counterfactual conditionals under both accounts. Even some proponents of the implicature view hold that “counterfactuality in *would*-conditionals, in general, cannot be canceled “for no reason”. Doing so would be a case of bad discourse manners” (Arregui and Biezma, 2016, p.6).

### 2.2. Conditional Perfection

The second inference that is relevant to our investigation is that of “conditional perfection” (Geis and Zwicky, 1971). As introduced above, CP can be derived *via* two different inference routes, either supplementing *if p, q* with *if not p, not q*, or with *only if p, q*. It is a cancelable inference (9-a) and does not arise in all conditionals to the same extent; conditionals resist perfection for instance in cases where world knowledge mandates that the consequent can also be obtained by means other than the ones stipulated in the antecedent (9-b). In consequence, the CP inference has been treated as a conversational implicature^1^.

(9) a. If you mow the lawn, I'll give you $5. But if you clean up the garage, I'll give you $5 too.
b. If you drink coffee, your heart rate may go up.

CP inferences are observable in a variety of speech acts (Searle, 1975, 1979) communicable by conditionals in indicative form, e.g., in assertive hypothetical conditionals (10-a), and in commissive conditional promises (10-b) and conditional threats (10-c), although there is an ongoing discussion about potential differences with regard to the respective prominence of the inference (Newstead, 1997; Evans and Twyman-Musgrove, 1998; Dieussaert et al., 2002; Zevakhina and Prigorkina, 2021). In the present article, we focus on hypothetical conditionals, therefore, we will not discuss these differences any further. Other types of indicative conditionals, however, are completely immune to CP. This includes biscuit conditionals (also called relevance conditionals or speech act conditionals) and premise conditionals (Franke, 2009; von Fintel, 2012). With respect to the former, it has been argued that they resist perfection due to the independence of the antecedent and consequent proposition (Franke, 2007, 2009; Lauer, 2015; Goebel, 2017). In (10-d), for instance, the biscuits on the sideboard continue to exist regardless of whether the addressee wants them. Therefore, both types of CP inferences (*if not p, not q*, or *only if p, q*) are invalid. With respect to the latter, CP is assumed not to arise in premise conditionals like (10-e) because of a presupposed belief in the antecedent proposition. This enforces an interpretation of the conditional with a focus on the consequences of the (presupposed) antecedent rather than the (sufficient or necessary and sufficient) conditions for the consequent, which may be incompatible with CP (see below for details).

(10) a. If the contract negotiations go well, Sue will take the job.
b. If you mow the lawn, I'll give you $5.
c. If you say one more word, I will kill you.
d. There are biscuits on the sideboard if you want them. (Austin, 1956)
e. A: John is in Amherst today.
B: If he is in Amherst, he be home late tonight. (von Fintel, 2001, p.18).

As outlined above, CP has been discussed extensively as a property of various types of indicative conditionals (among others, Atlas and Levinson, 1981; Newstead, 1997; Van Der Auwera, 1997; Evans and Twyman-Musgrove, 1998; von Fintel, 2001; Dieussaert et al., 2002; Franke, 2007; Van Canegem-Ardijns and Van Belle, 2008; Herburger, 2015; Lauer, 2015; Goebel, 2017). By contrast, CP in counterfactual conditionals has received less attention (but cf. Karttunen, 1971; Horn, 2000; Tellings, 2016). One of the reasons is that we seem to encounter a contradiction when trying to supplement counterfactual conditionals with the *if not p, not q* inference: due to inference to the falsity of the antecedent, (11-a) seems to presuppose that you did not mow the lawn, whereas (11-b) seems to presuppose that you did.

(11) a. If you had mowed the lawn, I'd have given you $5.
b. If you hadn't mowed the lawn, I wouldn't have given you $5.(Horn, 2000, p.320)

Intuitively, however, we can still derive a perfected interpretation *via* the *only if p, q* inference; this is what Horn (2000) proposes when arguing that counterfactual conditionals can be perfected much like indicatives, but require an evaluation of the inference from within the counterfactual world. That is, (11-a) is taken to implicate that in the counterfactual world in which I gave you $5, the only reason I would have done so is if and only if you mowed the lawn.

Similarly, Arregui and Biezma (2016) argue that counterfactual conditionals can still receive a perfected interpretation if they are understood as an exhaustive answer to the question about the conditions under which the consequent would have been obtained. This is related to von Fintel's (2001) account of conditional perfection, in which he argues that a conditional will receive a perfected interpretation if (and only if) it is understood as an exhaustive answer to a (potentially implicit) Question under Discussion (QUD) (Roberts, 2012) about the necessary conditions for the consequent proposition. Thus, a conditional that is understood as an answer to a question about the consequences of the antecedent (12-a) will usually not be perfected, whereas a conditional that is understood as an answer to the QUD in (12-b) can receive a perfected interpretation if it is taken to answer that question exhaustively.

(12) a. *QUD*: What (if anything) will happen if I mow the lawn?If you mow the lawn, I'll give you $5.
b. *QUD*: Under which conditions will you give me $5? If you mow the lawn, I'll give you $5.

Returning to counterfactual conditionals, we can now see Arregui and Biezma's (2016) reasoning: the pragmatic trigger for deriving a perfected interpretation in counterfactual conditionals is the same as in hypothetical indicatives, namely, the presence of a suitable implicit QUD. If that QUD is concerned with the counterfactual conditions under which the consequent would have obtained, the conditional can receive a perfected interpretation (13). Nonetheless, to our knowledge, it has so far not been empirically tested whether comprehenders really derive the CP inference to the same extent in hypothetical indicative and counterfactual conditionals.

(13) *QUD*: Under which conditions would you have given me $5? If you had mowed the lawn, I'd have given you $5.

Finally, the QUD approach offers a natural explanation as to why premise conditionals like (10-e) are imperfectible. As A's belief in the antecedent proposition is presupposed, B's response can only be interpreted as addressing a question about what may follow from said antecedent (von Fintel, 2001; Arregui and Biezma, 2016). Interpreting the conditional as the exhaustive answer to the conditions under which the consequent would obtain is infelicitous.

## 3. Attenuating NPIs in Conditionals

Having established the necessary background on pragmatic inferences in conditionals, we can now investigate how they affect the behavior of attenuating NPIs in conditional antecedents. The term attenuating NPI refers to a subtype of NPIs that is restricted to contexts in which they weaken the assertion they appear in (Israel, 1996), so that (14) with the attenuating NPI *all that*, e.g., is rather vague or uninformative about the kids' actual level of excitement for school (or lack thereof). Other examples of such NPIs include English *much* (Israel, 1996), German *sonderlich* (‘particularly’) (Schwab and Liu, 2022) and *so recht* (‘really’), and Japanese *anmari* (‘very’) (Matsui, 2013).

(14) The kids are^*^(n't) all that excited about school.

Existing accounts of NPI licensing, which have not specifically focused on attenuating NPIs, emphasize the role of veridicality (Giannakidou, 1998, 2006) or scalar properties of NPI and licensing context (among others, Ladusaw, 1979; Kadmon and Landman, 1993; Krifka, 1995; Chierchia, 2004, 2006). However, both types of accounts struggle to capture the full set of distributional restrictions on attenuating NPIs. For one, with respect to the latter type of accounts, they assume that NPIs are scalar operators which require a context in which they strengthen the assertion they appear in. Thus, for the indefinite NPI *anyone*, for instance, the assertion that *Mary did not see anyone* is more informative than alternatives in which *anyone* is replaced by a reference to a more specific set of individuals. Crucially, as argued above, the same does not hold for attenuating NPIs, as they weaken assertions they appear in.

More importantly, regarding both types of existing accounts on NPI licensing, Schwab and Liu (2022) made the novel observation that attenuating NPIs display an unexpected behavior with respect to their licensing in the antecedent of conditionals. The authors state that the English NPI *all that* and the German NPI *sonderlich* (‘particularly’) are degraded in the antecedent of hypothetical indicative conditionals (15-a) compared to the antecedent of counterfactual ones (15-b), and provide experimental evidence that quantitatively validates this pattern for the latter NPI. This finding goes counter to both scalar and veridicality-based accounts of NPI licensing. The former assumes that weak NPIs are licensed in contexts that are at least Strawson downward entailing, which holds for both hypothetical indicative and counterfactual conditionals (von Fintel, 1999). The latter assumes that weak NPIs are licensed by nonveridicality^2^ (Giannakidou, 1998, 2006), which, again, holds for both types of conditionals (in fact, indicative conditionals are nonveridical whereas counterfactual conditionals are antiveridical, which refers to a strict subset of nonveridical contexts in which the proposition *p* is entailed or presupposed to be false).

The assumption that both types of conditionals license weak NPIs has been motivated by the fact that this assumption does indeed hold for NPIs of the strengthening subtype, e.g., *any* or *ever* (15c-d). Schwab and Liu (2022), therefore, suggests that the pattern in (15a-b) is related to particular properties of attenuating NPIs.

(15) a. ?If the students have been all that attentive in class, they will pass the exam.
b. If the students had been all that attentive in class, they would have passed the exam.
c. If the students have paid any attention in class, they will pass the exam.
d. If the students had paid any attention in class, they would have passed the exam.

The peculiar behavior of attenuating NPIs motivates a novel theoretical analysis of the licensing mechanism for this NPI subtype, proposed in Schwab and Liu (2022). In the spirit of Israel (1996) and Krifka (1995), they propose a scalar licensing mechanism under which attenuating NPIs require that a more informative (i.e., stronger) alternative proposition is contextually available—where higher informativity is measured as the potential for the alternative proposition *p'* to add novel information to the common ground even after *p* has already been asserted. Alternative propositions are thought to be lexically evoked by the NPI; for a degree modifier NPI like *all that*, these are assumed to be propositions in which the degree argument specified by *all that*—which lexically designates that the modified predicate holds to a high degree—is replaced by a lower degree alternative^3^. Formally, this yields a licensing condition in which the asserted proposition *p* is put in relation to its evoked alternatives *p'*, such that the NPI is only licensed if there is an alternative *p'* that is more informative than *p* in terms of potential information gain via sequential contextual updates with *p* and *p'* (for full formal details, see Schwab and Liu, 2022)^4^.

Schwab and Liu show that, on this account, the degradation of hypothetical indicative conditionals falls out as a consequence of an interaction between the licensing mechanism above and the pragmatics of conditionals. Specifically, they emphasize the role of CP in indicatives and counterfactuals and the presupposed falsity of the antecedent in counterfactuals. With respect to the former, perfected conditionals do not license attenuating NPIs per the licensing mechanism introduced above. This is because neither the (perfected) conditional proposition *p* nor its (perfected) alternative *p'* is stronger than the other. Instead, the two propositions are mutually exclusive. To illustrate this, consider (15-a): under a perfected reading, (15-a) specifies that the necessary and sufficient degree of attentiveness for passing the exam is relatively high (exceeding a contextually supplied standard of attentiveness); all alternative propositions *p'*, which state that a lower degree of attentiveness is necessary and sufficient for passing the exam, must, therefore, be false and vice versa. Consequently, the degradation of attenuating NPIs in the antecedent of a conditional is assumed to be a function of the degree to which said conditional gives rise to a perfected interpretation. Interestingly, the same reasoning extends to the restrictor of universal quantifiers as licensing environment (as in *All students who have been (?all that) attentive in class will pass the exam*), in which case the restrictor can be strengthened into the necessary and sufficient condition for the consequent in the quantifier's scope (Schwab and Liu, 2022, p. 14).

With respect to the second inference, the presupposition of counterfactual conditionals is treated as a factor that can “rescue” (Giannakidou, 1998, 2006) the NPI even when its licensing is threatened by conditional perfection. Rescuing refers to a secondary licensing operation by which otherwise unlicensed NPIs can be salvaged if, within the context of the sentence, a licensing proposition is available for the NPI to associate with. The counterfactual conditional (15-b), e.g., presupposes *The students have not been all that attentive in class*, and this proposition in turn provides an environment in which the NPI *all that* is licensed.

Schwab and Liu note that their analysis raises two empirically testable questions. First, as mentioned in the previous section, there is uncertainty about whether CP emerges at all in counterfactual conditionals and, if so, whether it arises to the same extent as in hypothetical indicative conditionals. If the rate of CP is lower in counterfactuals, that would offer an immediate explanation for their increased acceptability as licensing environment for attenuating NPIs. Second, the theoretical analysis purports CP as a source of the degradation of conditionals. As such, it predicts that imperfectible conditionals will be a more acceptable licensing environment for attenuating NPIs. In the present article, we set out to test both of these questions. Across three behavioral experiments, we first replicate the degradation of the German attenuating NPI *sonderlich* in hypothetical indicative conditionals with the English attenuating NPI *all that*, then show that *all that* is improved in imperfectible premise conditionals, and finally determine that readers can draw CP inferences in both hypothetical indicatives and counterfactuals, although the inference rate may be reduced in counterfactuals. Altogether, our findings are mostly in line with the analysis proposed by Schwab and Liu. Moreover, they offer novel insight into the processing of presuppositions and implicatures in conditionals as well as the licensing mechanism of NPIs in general.

## 4. Experiment 1

The aim of the first experiment was to empirically verify Schwab and Liu's assumption that the English attenuating NPI *all that* is degraded in hypothetical indicative compared to counterfactual conditionals. To this end, we closely followed the methodology that Schwab and Liu employed to test the same phenomenon with the German NPI *sonderlich*. Using a graded rating scale, we asked English native speakers to indicate the naturalness of hypothetical indicative and counterfactual conditionals with or without the NPI *all that*.

Based on the aforementioned experiment in German, our hypotheses were that (a) hypothetical indicative conditionals with the NPI *all that* would be degraded compared to counterfactual ones, whereas (b) there would be no difference in the ratings for hypothetical indicative and counterfactual conditionals without NPI.

### 4.1. Methods

#### 4.1.1. Participants

For this and all subsequent experiments reported in this article, participants provided informed consent prior to participating. The experiments were approved by the ethics committee of the German Linguistic Society (Deutsche Gesellschaft für Sprachwissenschaft, DGfS). We initially recruited 77 participants *via* the crowd-sourcing platform *Prolific* (https://www.prolific.co/), two of whom were later removed due to low accuracy on comprehension questions (<80% correct responses across 36 *yes/no*-questions distributed over the filler items). All 75 remaining participants were English native speakers (47 women, 1 non-binary, aged 19–59, mean age = 34.8).

#### 4.1.2. Materials

In analogy to the experiment on German in Schwab and Liu (2022), we used 24 items in six conditions as shown in (17). Conditions (16a–d) were hypothetical conditional statements in indicative (16a,c) or counterfactual (16b,d) form. Conditions (16a,b) contained the NPI *all that* embedded within the antecedent, whereas conditions (16c,d) did not contain an NPI. Matching Schwab and Liu (2022), we also included two conditions using universal quantification, again either containing the NPI *all that* in the traditionally NPI-licensing restrictor of the universal quantifier (16e), or not containing an NPI (16f). These were included for exploratory purposes and will not be discussed at length (for a discussion of the relation between hypothetical indicative conditionals and universal quantifiers with regard to the licensing of attenuating NPIs, refer to Schwab and Liu, 2022). Participants saw only one of the six conditions per target item, pseudorandomly interspersed with 48 grammatical filler sentences so that two target items were always separated by at least one filler. The complete stimulus material, together with data and analysis code for all experiments, are available online (refer to “Data Availability Statement”).

(17) a. If the students have been all that attentive during class, they will pass the exam.
b. If the students had been all that attentive during class, they would have passed the exam.
c. If the students have been attentive during class, they will pass the exam.
d. If the students had been attentive during class, they would have passed the exam.
e. All students who have been all that attentive during class will pass the exam.
f. All students who have been attentive during class will pass the exam.

#### 4.1.3. Procedure

On each trial, a test sentence appeared in the middle of the screen. Participants were instructed to read the sentence and press the space bar once they were done. Upon doing so, a 7-point Likert scale would appear, asking participants to rate the sentence's naturalness from 1 (“completely unnatural”) to 7 (“completely natural”). Across all experiments, we opted for a 7-point Likert scale instead of a binary response option as the former is likely more sensitive for the detection and numerical estimation of small qualitative differences between conditions, particularly if participants' ratings for both conditions are located toward the same end of the scale (Schütze and Sprouse, 2013). In one half of the trials, distributed over the filler items, we subsequently asked participants to answer a *yes/no* comprehension question about the content of the sentence they had just read.

#### 4.1.4. Data Analysis

Data were analyzed with Bayesian ordinal regression models (Bürkner and Vuorre, 2019) using the *brms* package (Bürkner, 2017), version 2.12, in *R* (R Core Team, 2021), version 4.0. We ran two analyses, the first comparing hypothetical indicative and counterfactual conditionals (16a,c vs. b,d), and the second comparing universal quantifiers and hypothetical indicative conditionals (16a,c vs. e-f). The predictor variables *structure* (analysis 1: hypothetical indicative vs. counterfactual conditional; analysis 2: hypothetical indicative conditional vs. universal quantifier) and *NPI* (present vs. absent) were included as sum-coded fixed effects (0.5, –0.5) with an interaction term. We used the maximal random effects structure including random by-item and by-subject intercepts and slopes for all effects. We used the *brms* default priors, which were flat priors for the fixed effects and a Student's t-distribution with a mean of 0, 3 degrees of freedom, and a SD of 2.5 for the intercepts and random effects. Four chains were run with 8,000 sampling iterations each using a warm-up period of 4,000 iterations.

### 4.2. Results

**Figure 1:**
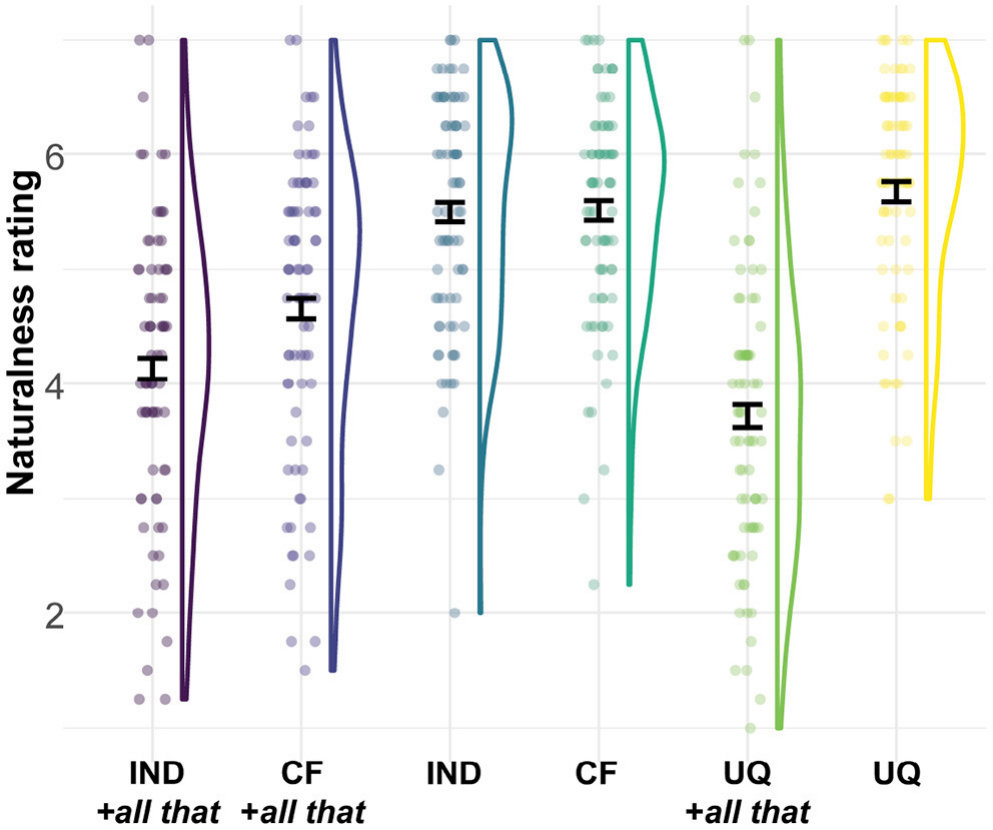
Naturalness rating on a 1–7 Likert scale for the six conditions of Experiment 1. The colored outlines show the distribution of the data, such that they are wider in places where more of the data is located. Dots show individual participants' mean rating across repeated measures. Error bars indicate the standard error around the mean. IND, indicative; CF, counterfactual; UQ, universal quantifier.

Observed naturalness ratings are visualized in Figure 1. The posterior estimates support an interaction effect between conditional form and NPI presence, such that with *all that* hypothetical indicative conditionals are less natural than counterfactual conditionals, whereas both types of conditionals are equally natural without the NPI, $\hat{\beta }$ = –0.48, CrI = [–0.81, –0.16], P(β <0) = 1. Even so, hypothetical indicative conditionals with attenuating NPIs are not rated as entirely unnatural.

For the second analysis, the posterior suggests an interaction effect, such that for both hypothetical indicative conditionals and universal quantifiers, sentences with *all that* are less natural than those without NPI, but the difference is more pronounced for universal quantifiers, $\hat{\beta }$ = –0.61, CrI = [–0.96, –0.25], P(β < 0) = 0.999.

### 4.3. Discussion

Our results closely match the predicted pattern, in that they resemble the German data in Schwab and Liu (2022). Schwab and Liu found that both types of conditionals were equally (highly) natural without the attenuating NPI *sonderlich* (‘particularly’), whereas hypothetical indicative conditionals with the NPI were degraded compared to counterfactuals. Our study finds the same pattern in English, thus replicating and extending the empirical evidence for the phenomenon observed by Schwab and Liu to a novel language and attenuating NPI.

One difference to note concerns the comparison between conditionals and universal quantifiers. Contrary to the German study, which indicated two main effects but no interaction in the comparison between hypothetical indicative conditionals and universal quantifiers with(out) the NPI, our study revealed an interaction effect in this regard. Indeed, in German, assertions with universal quantifiers were rated more natural than hypothetical indicative conditionals both with and without the NPI, whereas in English hypothetical indicative conditionals with *all that* were perceived as more natural than sentences using universal quantification. One speculative explanation is that the English materials using *all that* might have been more likely to trigger a premise conditional reading of the indicative conditional—in which case the analysis assumes that the NPI ought to be acceptable. Presumably, as *all that* can receive an anaphorical reading when stressed (e.g., *He's not all THAT stupid*, Onea and Sailer, 2013), participants may occasionally superimpose a covert preceding assertion onto the stimuli (e.g., *A: The students have been (very) attentive during class. B: If the students have been all THAT attentive [...]*). This would result in a premise conditional interpretation. In any case, interpreting the language differences with regard to the interaction effect is non-trivial, as it involves a comparison across two different statistical samples, languages, and linguistic constructions. The principal pattern, however, namely that hypothetical indicative conditionals and universal quantifiers with attenuating NPIs are degraded, has been confirmed by our study. This paves the way for two further experiments on the pragmatics of conditionals and their relation to the licensing of attenuating NPIs. As outlined in Section 3, Schwab and Liu (2022) predict that imperfectible indicative conditionals would be more acceptable as a licensing environment. We tested this prediction head-on with the following Experiment.

## 5. Experiment 2

In Experiment 2, we aimed to verify whether indicative premise conditionals with *all that* would be more acceptable than the hypothetical indicative conditionals tested in Experiment 1. If indeed the degradation of hypothetical indicative conditionals with *all that* is related to CP, imperfectible premise conditionals should provide a more acceptable licensing environment for the NPI. In addition, we tested what comprehenders assume about the epistemic state of a speaker who uttered a premise conditional with or without NPI. As noted in Section 3, the antecedent of indicative conditionals is (subjectively) nonveridical, i.e., the truth of the antecedent proposition is not entailed or presupposed. However, premise conditionals presuppose that somebody in the discourse believes the antecedent proposition. The speaker herself may accept that proposition, but can also employ the premise conditional as a rhetoric device to cast doubt on that belief (e.g., *A: Anna is in love. B: If she's really in love, she would have told us*.). Second, and independent of that, NPIs themselves have been shown to contribute a bias against *p* when occurring in the antecedent of hypothetical indicative conditionals (Liu, 2019). Thus, this raises the question of whether and to what extent comprehenders' beliefs about the veridicality of the antecedent proposition in hypothetical indicative conditionals and indicative premise conditionals (with or without NPI) differ.

Our hypotheses with respect to sentence naturalness were, therefore, that (a) hypothetical indicative conditionals with the NPI *all that* would be degraded compared to indicative premise conditionals, whereas (b) there would be no difference in naturalness for conditionals without the NPI. Moreover, with regard to assumed speaker belief in the antecedent, we predicted that comprehenders would attribute a higher belief in the antecedent proposition for premise conditionals than for hypothetical indicative conditionals due to the presupposition carried by the former. Based on Liu (2019), we predicted that the attributed speaker belief would be reduced in hypothetical indicative conditionals with the NPI than in ones without NPI. However, we had no clear predictions for an NPI effect in premise conditionals.

### 5.1. Methods

#### 5.1.1. Participants

We initially recruited 52 participants *via Prolific*, two of whom were later removed due to low accuracy on comprehension questions (< 80% correct responses across 25 *yes/no* questions distributed over the filler items). All 50 remaining participants were English native speakers (25 women, 25 men, aged 18–59, mean age = 37.4).

#### 5.1.2. Materials

We used 24 items in four conditions following a 2 × 2 design. All four conditions contained five sentences describing a miniature dialogue (18). The sentence of interest is the conditional uttered by the protagonist in the last sentence. In conditions (17a,b), provided the interlocutor's preceding utterance, it is a premise conditional echoing the presupposed information. In conditions (17c,d), it is a hypothetical indicative conditional. Conditions (17b,d) used the NPI *all that* embedded in the antecedent of the conditional, whereas conditions (17a,c) did not contain an NPI. Each item appeared with two questions, one asking for the naturalness of the last sentence, the other asking for the protagonist's belief in the antecedent proposition. Participants saw only one of the four conditions per item, pseudorandomly interspersed with 56 grammatical filler items that used similar miniature dialogues.

(18) Susan Smith works at a community college. / Her colleague says:
  a. “*The students have been attentive in class.”* / Susan Smith responds: / “*If the students have been attentive in class, they will pass the exam.”*
  b. “*The students have been very attentive in class.”* / Susan Smith responds: / “*If the students have been all that attentive in class, they will pass the exam.”*
  c. “*The students will start their exam season soon.”* / Susan Smith responds: / “*If the students have been attentive in class, they will pass the exam.”*
  d. “*The students will start their exam season soon.”* / Susan Smith responds: / “*If the students have been all that attentive in class, they will pass the exam.”*
  Q1: How natural was the last sentence?
  Q2: Does Susan Smith believe the students have been attentive in class?

#### 5.1.3. Procedure

Participants read the miniature dialogues one sentence at a time as indicated by the slashes in (18). The sentences appeared in the middle of the screen. Participants were instructed to read each sentence and press the space bar to proceed to the next one. Once they had read the last sentence, pressing the space bar would reveal the first question regarding the naturalness of the sentence they had just read. We used a 7-point Likert scale with endpoints marked as “completely unnatural” (1) and “completely natural” (7). Having answered that question, the second question regarding the protagonist's belief in the antecedent proposition would appear. Again, we used a 7-point Likert scale, this time with endpoints marked as “absolutely no” (1) and “absolutely yes” (7). In 25 trials, distributed across the filler items, the naturalness rating question was replaced by a *yes/no* comprehension question about the content of the miniature dialogue they had just read.

#### 5.1.4. Data Analysis

Data were analyzed with Bayesian ordinal regression models (Bürkner and Vuorre, 2019) using the *brms* package (Bürkner, 2017), version 2.12, in *R* (R Core Team, 2021), version 4.0. Responses to the two questions were analyzed separately but with identical model specifications. For both, the predictor variables *conditional* (hypothetical indicative vs. premise conditional) and *NPI* (present vs. absent) were included as sum-coded fixed effects (0.5, –0.5) with an interaction term. Random effects structures, priors, and several sampling iterations were the same as for Experiment 1.

### 5.2. Results

**Figure 2:**
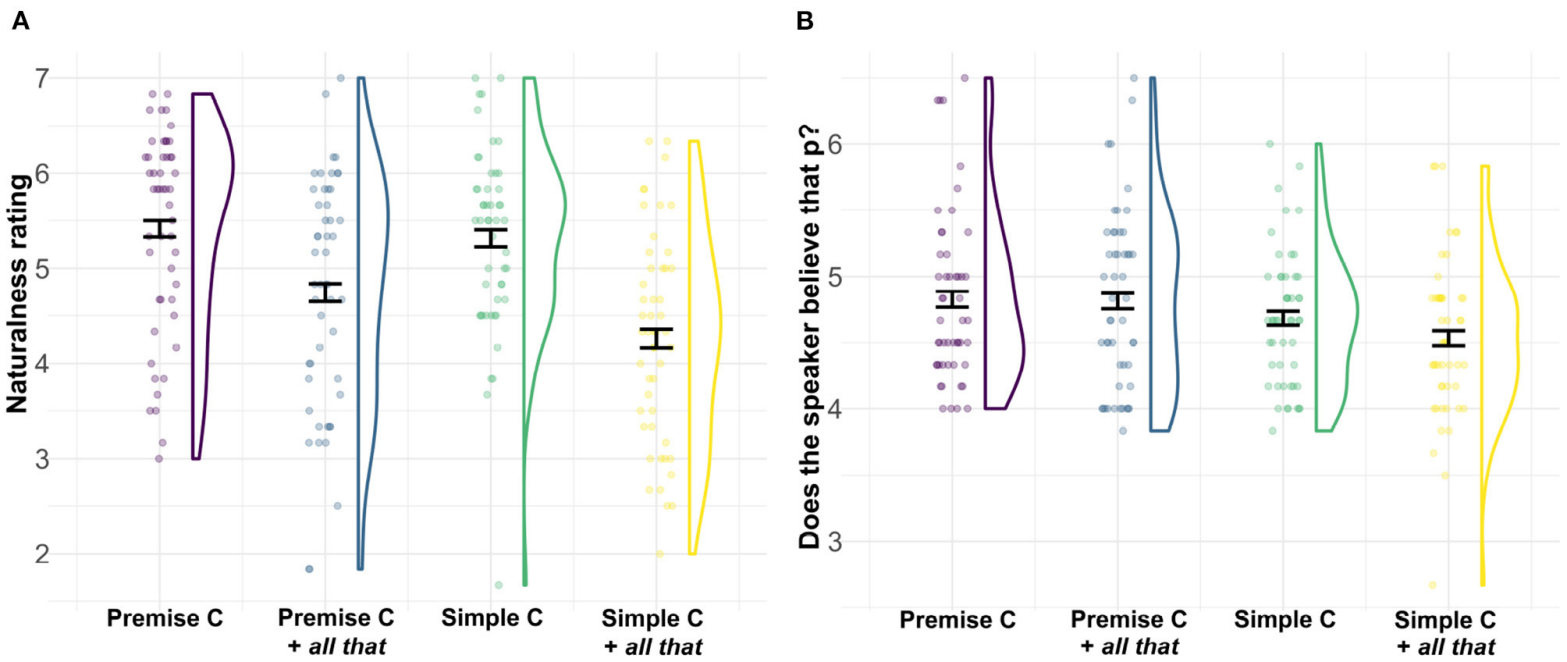
**(A)** Naturalness rating and **(B)** belief in the antecedent proposition on a 1–7 Likert scale for the four conditions of Experiment 2. The colored outlines show the distribution of the data, such that they are wider in places where more of the data is located. Dots show individual participants' mean rating across repeated measures. Error bars indicate the standard error around the mean. C, conditional.

Observed ratings on both questions are visualized in Figure 2. For the naturalness ratings, posterior estimates support an interaction effect, such that, without the NPI, hypothetical indicative conditionals and premise conditionals are similarly natural, but with the NPI, hypothetical conditionals are degraded compared to premise conditionals, $\hat{\beta }$ = –0.37, CrI = [–0.70, –0.05], P(*β* < 0) = 0.988.

For the belief in the antecedent proposition, the posterior suggests a main effect of *conditional* type, such that the speaker's belief in the antecedent proposition is perceived to be higher for premise conditionals than for hypothetical indicative conditionals, $\hat{\beta }$ = 0.30, CrI = [0.13, 0.47], P(β > 0) = 0.999. Additionally, the posterior is weakly supportive of an interaction effect, although the credible interval includes the zero point, thus leaving more uncertainty about the effect, $\hat{\beta }$ = –0.20, CrI = [–0.49, 0.09], P(*β* < 0) = 0.917. The interaction effect suggests that the speaker's belief in the antecedent is perceived to be reduced for hypothetical indicative conditionals with the NPI compared to those without, but is perceived as similar across the two premise conditionals.

### 5.3. Discussion

The results of the second experiment confirm our initial prediction that *all that* would be improved in the antecedent of premise conditionals. The findings are therefore in line with the proposal by Schwab and Liu. Additionally, as predicted, assessments of the perceived speaker belief show that participants interpreted premise conditionals as expressing a stronger commitment toward the truth of the antecedent proposition than hypothetical indicative conditionals. Note that this provides additional support for Schwab and Liu's argument that the degradation of indicative conditionals with *all that* is not due to the fact that the antecedent proposition is considered a possible state of the world—that is, the fact that indicative conditionals are nonveridical (but not antiveridical). On the contrary, our results show that *all that* can be licensed in premise conditionals, where *p* is as possible as in hypothetical indicative conditionals, if not even more likely.

Finally, our data matches the prediction that NPIs contribute a bias against *p* in hypothetical indicative conditionals (cf. Liu, 2019) but shows no such trend for premise conditionals. Liu (2019) assumes that the former (when using the conditional connective *if* ) are epistemically neutral—i.e., they do not convey any bias toward or against *p* (which is termed nonveridical equilibrium in (Giannakidou and Mari, 2021; Liu, 2021)). However, they can acquire a bias against *p* when combined with an NPI that conveys a weakened speaker commitment toward the antecedent proposition. Premise conditionals, on the other hand, are already biased toward *p* by virtue of their presupposition. This may cancel any weaker non-at-issue contributions of reduced speaker commitment by the NPI. It is beyond the scope of the current article to determine whether this pattern extends beyond the NPI *all that* or to other combinations of conventionally and conversationally commitment-conveying expressions.

To conclude, having seen the effects of the pragmatics of these two types of indicative conditionals on the inferred speaker belief and the acceptability of attenuating NPIs in the conditional antecedent, we turn to the second type of conditionals that is relevant within the theoretical analysis discussed in Section 3, namely counterfactual conditionals. Schwab and Liu (2022) and Experiment 1 showed that attenuating NPIs are more acceptable in the antecedent of counterfactual conditionals than hypothetical indicative conditionals. Schwab and Liu link this to either (a) reduced CP inferences, or (b) rescuing of the NPI in counterfactual environments. In the following experiment, we directly test how CP rates between hypothetical indicative and counterfactual conditionals differ, thereby providing empirical evidence to address option (a).

## 6. Experiment 3

The third and final experiment concerns a question raised in Section 2.2, namely whether comprehenders are equally likely to draw CP inferences from hypothetical counterfactual conditionals as from indicative ones. Although we have discussed the theoretical arguments in favor of CP in counterfactuals, we have also noted that it requires reasoning from within the counterfactual world. There is extensive psychological and cognitive-developmental work suggesting that processing hypotheticals, facts, and counterfactuals lead to differential engagements of brain regions (Nieuwland, 2012; De Brigard et al., 2013), and that counterfactual reasoning is particularly cognitively demanding (Riggs et al., 1998; Guajardo et al., 2009; Drayton et al., 2011; Van Hoeck et al., 2014, 2015). The latter has been attributed to the fact that it requires a dual representation of the actual and the counterfactual state of the world. A reasonable question is thus whether the increased cognitive demand of processing counterfactual conditionals may render comprehenders less likely to engage in the additional pragmatic processing required for inferences like CP.

Second, this experiment explores whether the addition of a degree modifier (like *all that*) in itself increases the CP rate in conditionals. Although this issue has not been raised in the analysis by Schwab and Liu (2022), it would further underpin why hypothetical indicative conditionals with *all that* are consistently degraded.

We hypothesize that CP inferences may arise more frequently if the antecedent contains a modifier because stating *if very p, q* over *if p, q* is at once weaker (as *very p* entails *p*, but not vice versa) and more costly to produce (as *very p* contains an extra modifier). Upon hearing *if very p, q*, comprehenders may thus infer that the unmodified variant does not hold, i.e., it is necessary and sufficient for the consequent that *very p*, not just *p*. Note that such competition with the unmodified variant is a typical effect with degree modifiers, including *very* and attenuating NPIs like *all that*, in scale-reversing contexts, often resulting in a duality in meaning. (19), e.g., allows for two readings, one in which there is uncertainty about the truth of the stronger proposition (*He's not bright)*, resulting in an attenuated interpretation, and one in which the stronger proposition is assumed to be true, but the speaker's choice of the more complex, weaker utterance is imparted additional meaning (Israel, 1996). In contexts involving sentential negation, this usually results in a negative understatement, such that the use of the negated modified predicate gives rise to an inference to its contrary (Horn, 1989; Israel, 2006).^5^ While this differs in some respects from the aforementioned inference in conditionals, it serves to demonstrate the general point that the use of the modified variant may impart the utterance with additional pragmatic meaning.

(19) He's not very/all that bright.
  a. Attenuation: He is (at most) moderately intelligent.
  b. Understatement *via* negative strengthening: He's really rather stupid.

Our predictions were, thus, as follows: (a) based on potentially increased processing demands for CP inferences from counterfactuals, we predicted that there would be fewer CP inferences from hypothetical counterfactual conditionals than from indicative conditionals. In addition, (b) we predicted that the CP rate from conditionals with a degree modifier in the antecedent would be higher than from antecedents with an unmodified adverbial. We did not predict an interaction between these two effects.

### 6.1. Methods

#### 6.1.1. Participants

We initially recruited 64 participants *via Prolific*, five of whom were later removed for failing attention checks. All 59 remaining participants were English native speakers (30 women, 2 non-binary, aged 18–64, mean age = 33.3).

#### 6.1.2. Materials

We created 24 items in eight conditions as shown in (20). In all conditions, three context-setting sentences preceded a critical conditional statement uttered by the protagonist. Conditions (19e-f) were included for the purpose of another experiment whose findings are not relevant to the research questions addressed in the present paper. For this reason, we will not discuss them any further but list them here for transparency about the experimental design. Conditions (19a–d) followed a 2 × 2 design with the factors conditional type and degree modifier presence. The conditional was presented in either indicative (19a,c) or counterfactual (19b,d) form, once with (19c,d) and once without (19a,b) the degree modifier *very* in the antecedent proposition. Contrary to Experiments 1 and 2, which included the degree modifier NPI *all that*, the present experiment used the polarity insensitive degree modifier *very*. Experiment 1 has already demonstrated that hypothetical indicative conditionals with the NPI *all that* are degraded. As in the present Experiment, we were not interested in sentence naturalness, but in inferences drawn from (natural) hypothetical and counterfactual conditionals, the inclusion of such a grammatically degraded construction could have confounded how participants respond to the inference questions. The change from using *all that* to using *very*, thus, ensured that all test sentences were equally well-formed. Each item appeared with two questions, the first asking about the protagonist's belief in the unmodified antecedent proposition, the second asking about the protagonist's belief in the *only if* conditional. We decided to assess CP through this inference route, rather than the *if not p, not q* inference, as the latter inference is complicated by the apparent clash between the presuppositions of affirmative and negated counterfactual antecedents (refer to Section 2.2). Participants saw only one of the eight conditions per target item, which were pseudorandomly interspersed with 56 grammatical filler items of similar length.

(20) Tom Scott loves watching movies and TV series. / New shows are coming out soon. / He says to his friend: /
  a. “*If the TV shows have been well written, they will receive positive reviews.”*
  b. “*If the TV shows had been well written, they would have received positive reviews.”*
  c. “*If the TV shows have been very well written, they will receive positive reviews.”*
  d. “*If the TV shows had been very well written, they would have received positive reviews.”*
  e. “*If the TV shows have not been well written, they will not receive positive reviews.”*
  f. “*If the TV shows had not been well written, they would not have received positive reviews.”*
  g. “*If the TV shows have not been well written, they will receive negative reviews.”*
  h. “*If the TV shows had not been well written, they would have received negative reviews.”*
  Q1: Does Tom Scott believe the TV shows have been well written?
  Q2 (conditions *a, c, e + g*): Does Tom Scott believe that the TV shows will **only** receive positive reviews if they have been (very) well written?
  Q2 (conditions *b, d, f + h*): Does Tom Scott believe that the TV shows would have **only** received positive reviews if they had been (very) well written?

#### 6.1.3. Procedure

The procedure was the same as for Experiment 2, except that both questions could be answered on a 7-point Likert scale with endpoints marked as “absolutely no” (1) and “absolutely yes” (7) and that we did not include comprehension questions.

#### 6.1.4. Data Analysis

Data were analyzed with Bayesian ordinal regression models (Bürkner and Vuorre, 2019) using the *brms* package (Bürkner, 2017), version 2.12, in *R* (R Core Team, 2021), version 4.0. Responses to the two questions were analyzed separately but with identical model specifications. For both, the predictor variables *conditional* (indicative vs. counterfactual) and *degree modifier* (present vs. absent) were included as sum-coded fixed effects (0.5, –0.5) with an interaction term. Random effects structures, priors, and the number of sampling iterations were the same as for Experiment 1.

Instead of comprehension questions, we used reading and reaction times as metrics for trial and participant exclusion. Prior to all other statistical analyses, we removed all trials in which one or more sentences within the trial (indicated by the time between space bar presses) were read in less than 100 ms (affecting 7% of all trials). As mentioned above, we also excluded five participants for whom more than 50% of trials were affected by the outlier removal process just described.

### 6.2. Results

**Figure 3:**
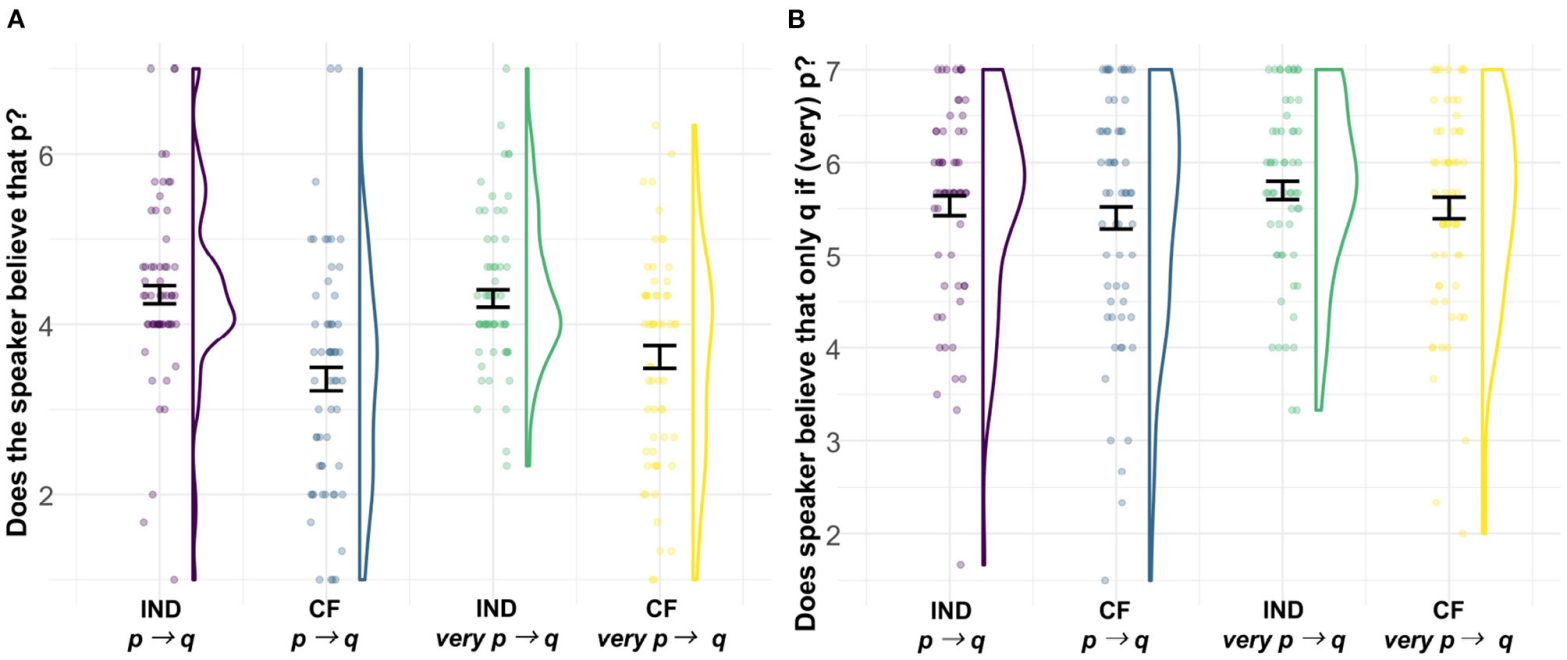
**(A)** Belief in the antecedent proposition and **(B)** belief in the inference to conditional perfection on a 1–7 Likert scale for the four conditions of Experiment 3. The colored outlines show the distribution of the data, such that they are wider in places where more of the data is located. Dots show individual participants' mean rating across repeated measures. Error bars indicate the standard error around the mean. IND, indicative; CF, counterfactual.

Observed ratings on both questions are visualized in Figure 3. For the first question—the speaker's belief in the antecedent proposition—the posterior supports a main effect of conditional type, such that the belief in the antecedent proposition is reduced for counterfactual conditionals compared to hypothetical indicative ones, $\hat{\beta }$ = 0.72, CrI = [0.47, 0.98], P(β > 0) = 1. There is no clear support for an effect of the degree modifier (P(β > 0) = 0.757) or for an interaction (P(*β* < 0) = 0.853).

For the second question—the speaker's belief in the *only if* conditional—the posterior is weakly supportive of two main effects (conditional type: $\hat{\beta }$ = 0.16, CrI = [–0.05, 0.36], P(β > 0) = 0.936; degree modifier: $\hat{\beta }$ = 0.13, CrI = [–0.09, 0.36], P(β > 0) = 0.885). These effects suggest that the belief in the *only if* conditional, i.e., the inference to conditional perfection, is endorsed less for counterfactual conditionals than for hypothetical indicative ones but is endorsed more if the antecedent proposition contains the degree modifier *very*. There is no support for an interaction effect, P(*β* < 0) = 0.548.

### 6.3. Discussion

With respect to comprehenders' judgments about the speaker's belief in the antecedent proposition, our results only partially align with standard assumptions about counterfactual conditionals. On the one hand, as predicted, they show that participants attribute a reduced belief in the antecedent proposition to the person uttering a counterfactual conditional. On the other hand, the observed responses indicate that participants are flexible in their interpretation of counterfactuals, such that the speaker is not always assumed to believe that the antecedent is false. This is in line with the theoretical claims made about the inference to the falsity of the antecedent on the basis of Anderson conditionals. Nonetheless, the rather high belief attribution was unexpected given that our stimulus material does not follow the typical Anderson-style structure, where the consequent proposition provides a reason why the falsity of the antecedent may not hold. One possible explanation is that our data is contaminated by participants who did not perform the experiment in good faith. We did not use comprehension questions to ensure attentiveness in this experiment, instead relying on reading times to exclude individual trials and participants. This may not have worked to a satisfactory extent. Another possibility is that participants were hesitant to make a categorical judgment on another person's (here, the speaker's) belief state given the limited context they were provided with. Participants do not know, e.g., whether Tom Scott in (20) has any particular insight on the TV industry which would allow him to conclude whether or not the upcoming TV shows have been well written. Finally, this result may in part be a quirk of English, where counterfactuality in conditionals is only marked with an additional layer of past morphology (cf. Iatridou, 2000; von Fintel, 2012). The counterfactual inference may be more salient in languages that instead employ grammatical markers of unreality, e.g., using subjunctive mood on the verb as in German (21).

(21) *Wenn die Fernsehsendungen gut geschrieben gewesen wären, hätten sie gute Rezensionen erhalten*.If the TV-shows well written had been SBJV had SBJV they good reviews received. “If the TV shows had been well written, they would have received positive reviews.”

More interestingly, our results also show that CP is present in both hypothetical indicative and counterfactual conditionals. With regard to hypothetical indicative conditionals, the high CP rate observed in the experiment is in line with previous findings for naturalistic linguistic stimuli (e.g., in Liu and Barthel, 2021). With regard to counterfactual conditionals, on the other hand, our study provides novel evidence that for these conditionals, too, comprehenders readily draw CP inferences. The hypothesis that CP would be reduced in counterfactuals due to increased processing demands could not be reliably confirmed, although our analysis indicates some tentative support for a small effect.

Finally, contrary to our prediction, the presence of a degree modifier in the antecedent did not reliably increase CP rates, although again the analysis indicates some support for a small effect in the expected direction. Note that, although the experiment reached a similar precision in the estimation of model parameters as the previous two experiments (indicated by CrI width), the estimates for the latter effects are smaller and their CrIs include both sides of 0. This indicates that the true effect size is either relatively small or that there is simply no true underlying effect, i.e., the null hypothesis is true. Our study did not have sufficient power to reliably detect such small differences; nonetheless, the posterior distributions attained for the effects can serve as valuable indicators of the effect sizes that can be expected in follow-up studies. To reliably confirm the effects, future replications that employ a sample size appropriate for a more precise estimation of parameter values are required.

## 7. General Discussion

Across three behavioral experiments, we have investigated the processing of NPIs and pragmatic inferences in conditionals. With regard to NPIs, in Experiment 1, we extended the previous findings that the German attenuating NPI *sonderlich* (‘particularly’) is degraded in hypothetical indicative conditionals to the English attenuating NPI *all that*. Experiment 2 confirmed a direct prediction by the proposed analysis in Schwab and Liu (2022), namely that the NPI would be improved in imperfectible conditionals. Finally, Experiment 3 demonstrated that CP inferences are readily made in counterfactual conditionals, suggesting that the improvement of counterfactual conditionals with *all that* is likely not attributable solely to differences in CP, but instead, as proposed in the analysis, requires rescuing *via* other means. Our findings, therefore, constitute the first empirical support from English for a theoretical account of the licensing of attenuating NPIs. Moreover, our findings indicate that pragmatic meanings in conditionals are computed rapidly enough to affect the licensing of NPIs embedded therein.

Our study looked at three different types of conditionals, with partially overlapping and partially distinct semantic and pragmatic properties. Despite their apparent similarity in form, hypothetical indicative conditionals and premise conditionals differ in that the latter presuppose the veridicality of the antecedent proposition in the discourse, a presupposition contributed by virtue of a preceding assertion. In contrast, counterfactual conditionals carry presuppositions about the falsity of the antecedent proposition, *via* counterfactual tense marking locally. Our study shows that both of these presuppositions modulate comprehenders' inferences about the speaker's belief in the antecedent proposition. However, our findings also suggest that such inferences about antecedent veridicality or antiveridicality are subject to further modulation, e.g., by the presence of NPIs or the assumed speaker's attitude toward the proposition.

Experiments 2 and 3 also demonstrated that presuppositions about the antecedent proposition affect whether comprehenders will draw an additional conversational implicature, CP. Interestingly, in one case (premise conditionals), CP is blocked, whereas in the other (counterfactual conditionals), CP is still available. One of the reasons may lie in the origin of the respective presupposition. On the one hand, in premise conditionals, the antecedent reiterates a previous speaker's assertion and thereby presupposes that some salient interlocutor believes that *p* is true. The resulting conditional can only be interpreted as an answer to a QUD about the consequences of the antecedent, rather than the (exhaustive list of) conditions for the consequent. Therefore, CP inferences do not arise (von Fintel, 2001; Arregui and Biezma, 2016). On the other hand, in counterfactual conditionals, the presupposition conveys that the speaker herself assumes that *p* is false. Crucially, *p* or *not p* need not have been asserted in the discourse context before. As such, counterfactual conditionals are compatible with an interpretation focusing on the latter type of QUD, resulting in CP. Nonetheless, counterfactual conditionals, too, can be coerced into a CP-blocking reading by preceding the counterfactual conditional with an *imagine-if* statement (22). This enforces a reading in which speaker *B* focuses on the consequences of the counterfactual possibility advanced by the antecedent, such that the CP inference becomes unavailable.

(22) A: Imagine if you had won the lottery!
B: If I had won the lottery, I would have quit my job.

An intriguing question that is beyond the scope of the current article concerns the mechanisms that subserve the incremental computation and integration of co-occurring types of pragmatic content in the processing of conditionals (and language in general). Future research may benefit from using temporally sensitive methods such as eye-tracking or EEG to elaborate on the processing of pragmatic meanings in conditionals, including presuppositions such as those carried by counterfactual and premise conditionals, and conversational implicatures such as conditional perfection.

## Data Availability Statement

The datasets presented in this study can be found in online repositories. The names of the repository/repositories and accession number(s) can be found below: https://osf.io/6zah8/.

## Ethics Statement

The studies involving human participants were reviewed and approved by the Ethics Committee of the German Linguistic Society (Deutsche Gesellschaft für Sprachwissenschaft, DGfS). The patients/participants provided their written informed consent to participate in this study.

## Author Contributions

JS and ML contributed to the conception and design of the studies. JS implemented the studies, performed data collection, statistical analyses, and wrote the first draft of the manuscript. ML provided funding acquisition, project administration, and resources. Both authors contributed to manuscript revision, read, and approved the submitted version.

## Funding

JS gratefully acknowledges support from the Deutsche Forschungsgemeinschaft (DFG, German Research Foundation), Research Training Group Computational Cognition (DFG-GRK 2340). ML's s work was funded by the DFG—SFB 1412, 416591334; SPP 1727, 367088975.

## Conflict of Interest

The authors declare that the research was conducted in the absence of any commercial or financial relationships that could be construed as a potential conflict of interest.

## Publisher's Note

All claims expressed in this article are solely those of the authors and do not necessarily represent those of their affiliated organizations, or those of the publisher, the editors and the reviewers. Any product that may be evaluated in this article, or claim that may be made by its manufacturer, is not guaranteed or endorsed by the publisher.

## Footnotes

^1^It is a separate and controversial question of how to treat this implicature formally. Proposals have ranged from treating it as scalar implicature (Van Der Auwera, 1997), Q-based implicature (Horn, 2000), R-based implicature (Atlas and Levinson, 1981), or quantity implicature based on the exhaustivity of answers (von Fintel, 2001). Refer to Herburger (2015) for an overview.

^2^Subjective nonveridicality is defined as such: A propositional operator *F* is veridical iff *F*_*p*_ entails or presupposes that *p* is true in some individual's epistemic model *M*_*E*_(*x*); otherwise *F* is nonveridical (Giannakidou, 2006, p. 589).

^3^With respect to (14), alternatives *p'* are thus propositions such as *The kids aren't (the least bit/somewhat) excited about school*, as well as the unmodified proposition, in which case the degree argument specifies the contextual standard of *excitement*.

^4^Formally, this yields the following licensing condition, where *p* refers to the asserted proposition with NPI and *p'* to its evoked alternatives. Within its first conjunct, the assertability/licensing condition below states that the proposition *p* is true. In its second conjunct, it states that there exists an alternative *p'* from the set of alternatives of *p* (evoked by the NPI), such that the alternative *p'* is compatible with the current context and such that updating the context c with *p* and subsequently (Strawson) updating with *p'* would have not yielded the same outcome as only updating with *p*. That is, *p'* removes some additional words which are compatible with p, and, therefore, would have been more informative than only asserting *p*:

(i) **Licensing condition**: $\left\{w\in c|w\in {\left[\left[p\right]\right]}_{c}\wedge \exists p^{\prime }\in Alt\left(p\right)\left(\exists w^{\prime }\in c|w^{\prime }\in {\left[\left[p^{\prime }\right]\right]}_{c}\wedge c+p{+}_{str}p^{\prime }\neq c+p\right)\right\}$ (Schwab and Liu, 2022).

^5^The speaker's choice of the modified variant might not only reflect their uncertainty or politeness (Gotzner and Mazzarella, 2021) but also their registered knowledge, i.e., knowledge about the situational-functional setting of the conversation.
